# Mariculture Probiotics: A Scoping Review of Research Trends, Knowledge Gaps and Opportunities

**DOI:** 10.1007/s10126-026-10701-7

**Published:** 2026-09-04

**Authors:** Lucien Alperstein, Jadranka Nappi, Suhelen Egan

**Affiliations:** Centre for Marine Science and Innovation, School of Biological, Earth and Environmental Sciences, Faculty of Science, The University of New South Wales, Room 501B, Level 5 East, Biological Sciences North (D26), UNSW, Kensington, NSW 2052 Australia

**Keywords:** Biotechnology, Microbiome manipulation, Beneficial microorganisms, Mariculture, Probiotics

## Abstract

The use of probiotics – live microorganisms – to enhance growth and prevent disease in mariculture species is an emerging alternative to conventional methods including antibiotics, vaccines and chemical intervention. While others have reviewed specific host organisms and probiotic genera, this review synthesises the scientific literature on the use of probiotics across a range of marine organisms to identify knowledge gaps and research opportunities. We identified widespread methodological heterogeneity, making direct inter-study comparisons difficult. Despite this limitation, 86% of in vivo studies documented positive outcomes following probiotic administration. Crustacean and finfish research dominates the field, encompassing 77% of the current literature, while molluscs, macroalgae and other invertebrates remain relatively understudied despite their significant ecosystem services and contribution to aquaculture. Probiotics provide multiple reported benefits to marine hosts, including increased growth and survival, and protection against pathogens. *Bacillus* and *Lactobacillus* from terrestrial environments are the dominant probiotics used across all studies, while marine-derived probiotic genera including *Pseudoalteromonas*, *Vibrio*, *Phaeobacter*, *Shewanella*, and *Halomonas* showed benefits across several hosts but remain relatively underutilised. Approximately 34% of publications evaluated the use of microorganisms isolated from the same host species in which the probiotics were tested. This review highlights the opportunity for further research into underutilised autochthonous probiotic genera and development of standardised research guidelines. We propose that future research should prioritise understanding probiotic mechanisms of action, and their use in rapidly expanding and understudied systems including macroalgae and molluscs, to ensure continued productivity with minimal impact on marine ecosystems.

**Supplementary Information:**

The online version contains supplementary material available at https://doi.org/10.1007/s10126-026-10701-7.

## Introduction

Marine organisms including finfish, shellfish and macroalgae (seaweed) provide food, pharmaceuticals, industrial products, animal feed and fertiliser (Leandro et al. 2019, Francesch et al. 2024). However, marine ecosystems are under threat from ocean warming, pollution, and resource overuse (Hoegh-Guldberg and Bruno 2010, Agostini et al. 2021, Halpern et al. 2015). Fisheries and aquaculture produce over 230 million tonnes of aquatic biomass annually, with aquaculture surpassing wild-catch fisheries in production for the first time in 2022 (Food and Agriculture Organization of the United Nations 2024c). As productive land becomes scarcer, marine aquaculture (mariculture) continues to expand faster than terrestrial agriculture (Little et al. 2016). Maintaining healthy oceans is therefore critical to sustain both aquaculture and wild-catch fisheries to meet the 22% growth required to feed the projected 1.7 billion additional people living by 2050 (Food and Agriculture Organization of the United Nations 2024c).

Disease has posed a major challenge to aquaculture since production intensification in the 1980s. High stocking densities, poor biosecurity, contaminated equipment and global trade have all led to frequent and damaging outbreaks (Bondad-Reantaso et al. 2023, Leung and Bates 2013, Dayana Senthamarai et al. 2023, Rodger 2016, Austin 2023). Antibiotics, used prophylactically to prevent or control bacterial disease outbreaks or to prevent secondary bacterial infections associated with viral diseases, can disrupt marine microbial and algal populations (Bondad-Reantaso et al. 2023, Schar et al. 2020, Cherian et al. 2023) and contribute to the emergence of antimicrobial resistance (AMR) in aquaculture pathogens (Adenaya et al. 2023). AMR genes can transfer to human pathogens (Pepi and Focardi 2021), posing risks to human health, especially where the same antibiotics are used in aquaculture and in human medicine. Given the efforts to restrict antibiotic and harmful chemical use (Bondad-Reantaso et al. 2023, Fitridge et al. 2012), there is an urgent need for alternative solutions to improve growth, minimise disease and increase sustainability practices. Vaccination has emerged, particularly in finfish, as a promising alternative to antibiotics in combatting disease (Tammas et al. 2024, Ma et al. 2019). However, vaccine use is, in some cases, difficult or inefficient. Many finfish can only be vaccinated once they have reached a large enough size (Dalmo et al. 2016), most existing vaccines require multiple boosters or cold-chain transport (Tammas et al. 2024), and current vaccine methods that rely on an adaptive immune response are not suitable for crustaceans, molluscs or algae (Guo et al. 2015).

Recent attention has therefore shifted towards the microbiome – the community of microbial symbionts living in and on host organisms – as a key factor influencing health, development, and disease resistance in aquaculture species (Diwan et al. 2023, Lorgen-Ritchie et al. 2023, Luan et al. 2023, Xavier et al. 2024, Sehnal et al. 2021, Alsufyani et al. 2020). The recognition of the importance of host microbiomes has driven renewed interest in using microorganisms, or probiotics, to manipulate health outcomes in a wide range of mariculture hosts as an alternative to antibiotics or chemical intervention. For the purposes of this review, we use the commonly cited joint United Nations Food and Agriculture Organization (FAO)/World Health Organisation definition of probiotics as “Live microorganisms which when administered in adequate amounts confer a health benefit on the host” (Food and Agriculture Organization of the United Nations and Organisation 2002).

In marine systems, probiotics are reported to confer a range of health benefits (as reviewed in (Knipe et al. 2021, Čanak et al. 2024, Simón et al. 2021, Peixoto et al. 2017, Waiho et al. 2025, Calcagnile et al. 2024, Zorriehzahra et al. 2016)) including mitigation of heavy metal toxicity (Abdel-Tawwab et al. 2024), increased growth (Javadi and Khatibi 2017, Sadat Hoseini Madani et al. 2018), survival (Javadi and Khatibi 2017, Adorian et al. 2019) and disease prevention (Castex et al. 2008, Chiu et al. 2007). Probiotics may boost immune and digestive function (Sadat Hoseini Madani et al. 2018, Adorian et al. 2019, Chiu et al. 2007) and improve water quality (Vadstein et al. 2018, Truong et al. 2022, Rahayu et al. 2024). These benefits mirror well-studied benefits of probiotics in terrestrial systems, where probiotics can improve human health (Hemarajata and Versalovic 2013, Valdes et al. 2018, Gupta et al. 2016), and both reduce disease burden and increase productivity in agricultural crops (Gavelienė and Jurkonienė 2022, Jiménez-Gómez et al. 2017, Jaiswal et al. 2021, Avis et al. 2008, Bakhshandeh et al. 2020).

At present, off-the-shelf probiotics consisting of terrestrial microorganisms are often used in marine aquaculture (for successful examples see (Abdel-Tawwab et al. 2024, Javadi and Khatibi 2017, Sadat Hoseini Madani et al. 2018, Adorian et al. 2019, Castex et al. 2008, Vadstein et al. 2018)). However, the microbiomes of marine organisms differ to those of terrestrial plants and animals (Luan et al. 2023, Kim et al. 2021), and the long-term safety of allochthonous (non-native) probiotic use in marine organisms remains unknown (Holt et al. 2021). In addition, administering probiotics to marine organisms poses unique challenges, where biofilm dynamics (Wahl et al. 2012), modes of disease transmission (McCallum et al. 2004), host life histories and reproductive strategies can differ from their terrestrial counterparts (Steele et al. 2018). Therefore, autochthonous marine microorganisms, i.e., those associated with the host they are intended to be used in, should be further explored for use in marine organisms (Doan et al. 2020).

Despite their potential benefits knowledge of marine microbiomes remains limited compared to terrestrial and freshwater aquatic hosts. Disease mechanisms are often poorly understood, and relatively few studies have explored probiotic interventions in marine species (however see (Hudson and Egan 2024, Ou et al. 2021, Egan and Gardiner 2016)). Addressing these knowledge gaps is key for the development of novel biotechnologies that will support sustainable marine aquaculture growth. While previous reviews in this area have focused on specific host taxa (Knipe et al. 2021, Sumon et al. 2022, Yeh et al. 2020), microbial genera (Gabadage et al. 2023, Vasyliuk et al. 2023, Hariharan and Dharmaraj 2018, Das et al. 2008), or methods used to identify new probiotics (Calcagnile et al. 2024), a synthesis across all taxa specifically in the marine environment is missing. To better understand the research landscape and identify critical knowledge gaps in probiotic research, we conducted a scoping review of existing literature on probiotic research in marine organisms, covering species of both ecological and mariculture relevance. Specifically, we analysed the literature to determine:

- Whether publications were in vitro studies only or involved in vivo intervention and/or in vivo challenge experiments.
- The countries where marine probiotic research is being conducted.
- The breadth of marine hosts in which specific microorganisms are being tested for probiotic effects (i.e., as probiotics).
- The positive effects for which probiotics are being tested.
- The diversity and origin of probiotic microorganisms.
- The challenges and opportunities for marine probiotic development.

## Methods

### Literature Search, Inclusion Criteria and Data Collection

Peer-reviewed primary literature was retrieved using Scopus, PubMed and Web of Science (Core Collection) with publication date set up to 25 May 2025 and records limited to title/abstract/keywords using the following search terms:

[(“microbio*” OR “probiotic*” OR “bacteri*” ) AND ( “marine” OR “ocean” OR “sea*” OR “reef”) AND ( “seaweed*” OR “fish*” OR “finfish*” OR “macroalga*” OR “alga*” OR “prawn*” OR “shrimp*” OR “crustacea*” OR “mollusc*” OR “aquaculture” OR “seagrass*” OR “coral*” OR “lobster*” OR “crab*” OR “urchin*” OR “sea cucumber*” OR “sponge*” OR “cnidaria*” OR “mussel*” OR “oyster*” OR “abalone*”) AND ( " manipulation " OR " augmentation " OR " addition " OR " dysbiosis” OR “morphogenesis” OR “probiotic” OR “synbiotic”) AND NOT ( “human” OR “mice” OR “mouse” OR “murine” OR “freshwater” )]. Relevant primary literature identified from review articles published between May 2021 and May 2025 were also included. Grey literature was not considered for this study as we wanted to evaluate peer-reviewed published scientific research only.

The Rayyan review management program (https://www.rayyan.ai) was used to remove duplicates.

Unique publications were manually screened for inclusion according to the following criteria:

Publications were included if they met the following criteria:

i. Involved the isolation and/or screening of microorganisms against known pathogens of marine organisms in vitro or tested bile tolerance or gut colonisation of microorganisms, or,
ii. Evaluated isolated or previously cultured microorganisms in vivo in marine hosts to test for probiotic effects.
iii. Were published in English or provided an English translation.

Publications were excluded if they:

i. Were unrelated to marine hosts or hosts were microscopic organisms (e.g., rotifers). Publications with anadromous animals were included if the growing conditions were saline. Our intention was to analyse marine organisms specifically and so freshwater hosts were not included in this study.

Studies identified within included publications were categorised into the following study types:

1. **In vitro *s*tudies**. Studies that used probiotics to antagonise, outcompete or reduce the virulence of known or suspected pathogenic microorganisms. Host organisms were listed for this category if the authors explicitly mentioned an intended host, even though the probiotics were not tested in vivo in that host.
2. **In vivo intervention studies**. Studies that used probiotics to enhance the growth, morphology, survival, disease resistance, stress responses, immune responses, enzyme production, or impart other positive outcomes to a host organism.
3. **In vivo challenge studies**. Studies where probiotics were used alongside known or suspected challenge conditions, including administering of pathogenic microorganisms or physical challenge conditions (such as elevated water temperatures). In these studies, probiotics could be administered before, concurrently or after the pathogens/stress conditions.

If publications contained components of multiple study categories (e.g., in vitro testing and an in vivo challenge trial), the publication was categorised into the more advanced study type only (i.e., in vivo challenge > in vivo intervention > in vitro).

The following data was collected from each publication: publication year, host system, if the probiotic(s) tested were isolated in the study, the source of the probiotic, the study type (as defined previously in this section), the host organism(s), the host organism’s life stage (e.g., larva, juvenile, adult), the probiotic(s) tested, the pathogen(s) tested against (where applicable), the duration of the study, how the probiotic(s) were administered, if the study used multiple probiotics or other adjuvants in addition to probiotics (e.g., prebiotic supplements, microalgae). Where a publication did not list the microbial composition of commercial probiotics, we identified these details from grey literature where possible.

For in vivo intervention and in vivo challenge trial studies, data were collected for growth/weight gain, reproduction, survival, disease prevention/treatment, microbiota analysis, lower pathogen count/competitive exclusion (based on the definition from (Levin 1970)), immune markers (e.g., increase/decrease in haemocytes, phenoloxidase, superoxide dismutase) and digestive enzymes (e.g., increase/decrease in protease, lipase, carbohydrase) and a catch-all “other” category for other factors. For each of these categories, statistically significant positive, negative, neutral or unclear results were recorded. We also collected data for in vitro inhibition results where microorganisms were tested against pathogens, the country where the study was conducted and whether the probiotic(s) had been isolated from the same host organism species in which it was tested.

### Data Analysis and Visualisation

Data were compiled in Microsoft Excel (version 2505) and analysis conducted using the dplyr package in RStudio (RStudio 2023). Data visualisation was conducted using ggplot2 with accessible colour scheme *viridis*. ChatGPT-4.5 (OpenAI 2025) was used as per journal guidelines to assist in editing data visualisation scripts, with content manually reviewed for accuracy.

## Results

### Scope of the Literature

Database searches identified 5 038 (Scopus), 3 653 (PubMed), and 8 812 (Web of Science) articles. After duplicate removal and manual screening, 892 publications met the inclusion criteria.

**Fig. 1:**
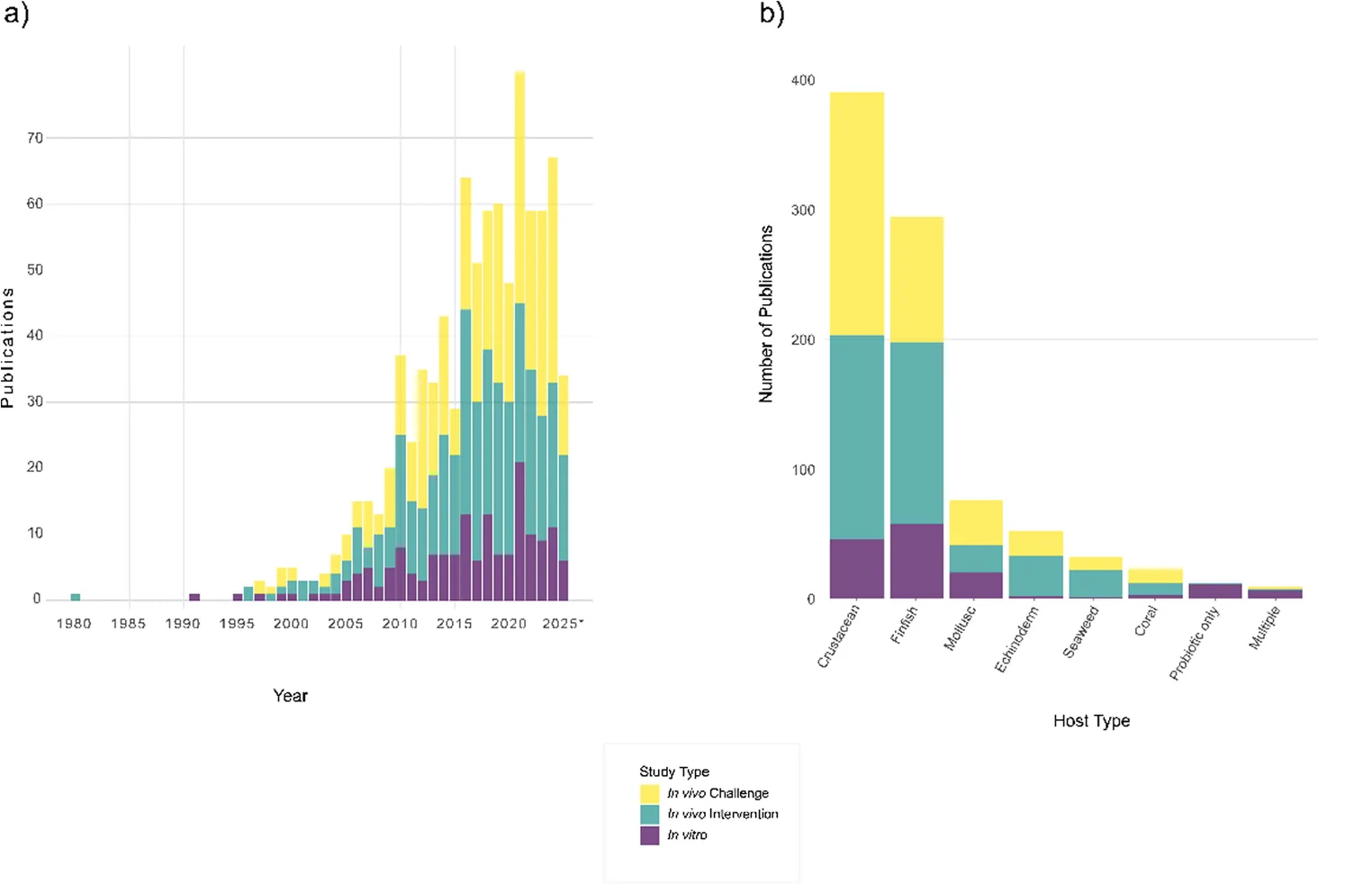
**a**: The number of publications investigating probiotics in marine hosts conducted per year by study type, with in vitro, in vivo intervention and in vivo challenge studies coloured. Of the 892 publications, 142 were in vitro studies only, 315 in vivo intervention only, 248 in vivo challenge only, 72 in vitro and in vivo intervention and 115 in vitro and in vivo challenge. *Data for 2025 is to 25 May 2025 only. **b**: Number of publications categorised by study type for different host organism types, with in vitro, in vivo intervention and in vivo challenge studies coloured as per figure legend. Where publications contained multiple study types, they were categorised into the more advanced category for both 1a and 1b (i.e., in vivo challenge > in vivo intervention > in vitro). Studies that tested probiotics in multiple host types were placed into the “multiple” category

Publications on the use of probiotics in marine organisms increased over time (Fig. 1a), with a peak of 80 publications in 2021 and the proportion of any one study type (in vitro, in vivo intervention, and in vivo challenge) remaining relatively stable over time.

Publications that conducted in vivo intervention and challenge studies were more common than in vitro only studies. Publications conducting in vivo challenge studies were more likely to also include in vitro components (e.g. (Sha et al. 2016)), compared to in vivo intervention publications (e.g. (Liñan-Vidriales et al. 2021)), .

### Geographic Distribution of Research Outputs

Publications were conducted in 47 countries (Supplementary Fig. 1), with 22.9% from China (205 publications), followed by Spain (61 publications) and India (59 publications). Regionally, Asia contributed 472 publications, Europe 169, the Middle East 49, the Americas 145, Africa 30 and Oceania 27.

Over 90% of aquaculture products are produced in Asia (Food and Agriculture Organization of the United Nations 2024c) but only 52.9% of research publications were conducted in the same region. In contrast, Europe produces only 2.7% of global aquaculture products (Food and Agriculture Organizationof the United Nations 2024c, Food and Agriculture Organization of the United Nations 2024a) but produced 18.9% of the published research.

### Host Type and Relationship Between Research Output and Aquaculture Value

Publications were grouped into six host categories (Fig. 1b) based on the International Standard Statistical Classification of Aquatic Animals and Plants (Food and Agriculture Organization of the United Nations, 2000).

Nearly half of all publications (401) evaluated probiotics in crustaceans, particularly white leg shrimp (*Litopenaeus vannamei -* formerly *Penaeus vannamei*, 251 publications). Finfish were the second most studied category (303 publications) with *Sparus aurata* (38), *Dicentrarchus labrax* (34), *Scophthalmus maximus* (21) and *Solea senegalensis* (16) used most frequently. Fewer publications focused on molluscs (77), echinoderms (53), seaweeds (33) or corals (24) (Fig. 1b). Nine publications were conducted on multiple host species (e.g., finfish and crustacea, finfish and seaweed, and are included above in individual host totals) and twelve did not specify a host.

Host type appeared to influence whether studies combined multiple probiotic treatments or conducted challenge trials (Fig. 1b). Studies on crustaceans, finfish, molluscs, corals and echinoderms included more complex experimental designs, such as multiple probiotic species, consortia, or challenge trials, while most seaweed studies were typically limited to laboratory-scale trials assessing restoration of growth in axenic cultures.

Research effort, measured by publication output, appears to differ from patterns of aquaculture production and economic value across sectors (supplementary table S1, based on UN FAO aquaculture production and value data (Food and Agriculture Organization of the United Nations 2024a, Food and Agriculture Organization of the United Nations 2024b)). Molluscs and seaweeds together accounted for approximately 78% of production by weight and 38% by value but represented only 12.3% of probiotic publications. In contrast, crustaceans accounted for 10.9% of production by weight and 31.1% by value yet represented 45% of probiotic publications.

### The Same Probiotic Genera Genefit Multiple Hosts

**Fig. 2:**
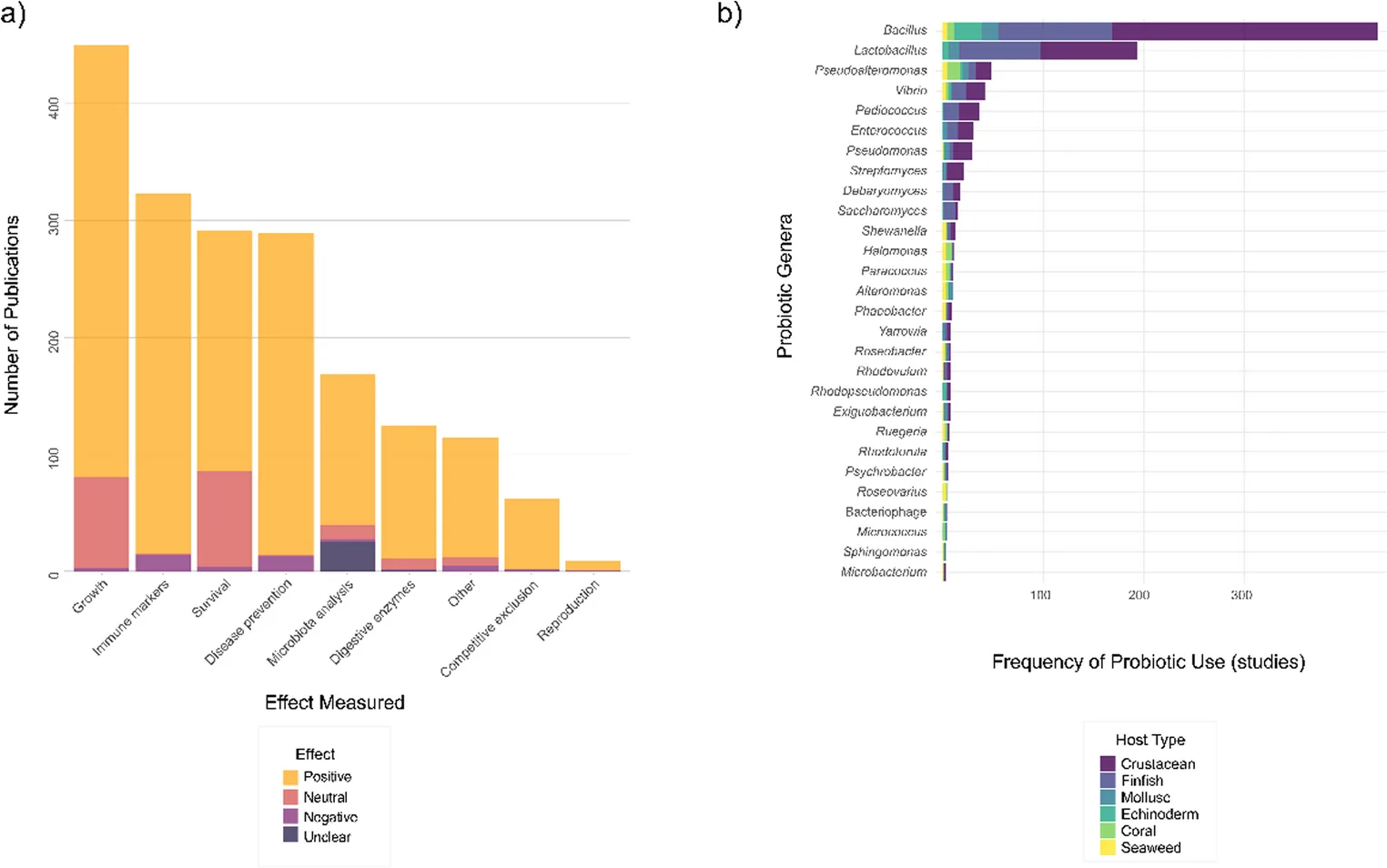
**a**: Total number of publications testing different probiotic effects for all host types, according to effect on the host (i.e., positive, negative, neutral or unclear). **b**: Total number of probiotic genera and host types used in in vivo intervention and challenge studies within publications, for probiotic genera used in 3 or more different host types. Each bar represents the total number of studies in which a probiotic genus was used, with colours corresponding to different host types. Probiotics were counted multiple times in publications where they were evaluated in multiple host species or against different pathogens. *Note that consortia are included as a single entry representing all combinations of consortia used. See supplementary Excel file, Table 3, for details of consortia

Host organism growth, immune markers, survival and disease prevention were the most frequently measured probiotic effects (Fig. 2a, and supplementary Excel file, Table 4). Other less frequently reported probiotic effects include those related to reproduction, water quality and competitive exclusion of pathogens. Across all publications testing probiotics in vivo, 85.7% of reported outcomes were positive, 11.8% neutral, 1.0% undesirable and 1.5% undefined or unclear (such as changes in the microbiota under different treatments that may have correlated with positive outcomes, but without a link to a specific benefit e.g. (Liñan-Vidriales et al. 2021, Zhang et al. 2023)).

A total of 174 microbial genera were tested for probiotic activity in vivo. Fifty-two of these genera were tested for beneficial activity in more than one host and 28 were tested in 3 or more host types. *Bacillus* and *Lactobacillus* were used most frequently in in vivo studies, together found in 44% of all publications (290 and 147 publications respectively, and over 40 studies using both genera), in 433 and 196 studies respectively, and used in six different host types (see Fig. 2b for a subset of probiotic genera used, and supplementary Excel file, Table 2, for a complete table). *Bacillus subtilis* was used most in in vivo studies (136 studies), followed by *Lactobacillus plantarum* (72 studies), *Bacillus licheniformis* (57 studies) and *Lactobacillus acidophilus* (17 studies). These two genera were also more likely to have been derived from terrestrial sources compared to other probiotics (35.5% vs. 13.2% for other probiotic genera). *Vibrio* isolates had probiotic effects in over 50 in vivo studies. Other microbial genera tested in four or more host types, albeit less frequently, were *Pseudoalteromonas*, *Pseudomonas*, *Shewanella*, *Alteromonas*, *Paracoccus*, *Phaeobacter*, *Roseobacter* and *Psychrobacter*.

In vivo challenge trials typically sought to reduce disease or mortality induced by *Vibrio* species including *V. alginolyticus*,* V. harveyi*,* V. parahaemolyticus and V. campbellii*. Sixty-one probiotic genera were effective at reducing *Vibrio*-induced disease or mortality across four different host types (e.g., (Sha et al. 2016, Vogeley et al. 2019, Ravi et al. 2007, Kesarcodi-Watson et al. 2009, Sohn et al. 2016)) . Many probiotic genera were also effective against a range of bacterial pathogens including *Aeromonas* sp (Paralika et al. 2023, Wang et al. 2019), *Photobacterium* sp (Peixoto et al. 2018) and *Pseudoalteromonas* sp (Li et al. 2022, Hjelm et al. 2004).

Probiotic consortia were tested in 184 publications. Approximately 95% of these publications reported a benefit to the host, including traits such as improved growth, survival, and/or immune responses.

### Source of Probiotic Isolates

Of the 892 publications, 33.6% (300) used autochthonous microorganisms isolated from the same host species being tested. For the remaining publications, probiotics were derived from a variety of sources, including marine and freshwater eukaryotes, marine sediment, faeces, fermented food products, aquaculture ponds and seawater (see supplementary Excel file, Table 1).

Publications on corals, seaweeds, and molluscs were most likely to use autochthonous microorganisms (91.3%, 71.9%, 51.9%, respectively), while finfish and crustaceans were least likely (30.7% and 24.3%, respectively).

## Discussion

**Fig. 3:**
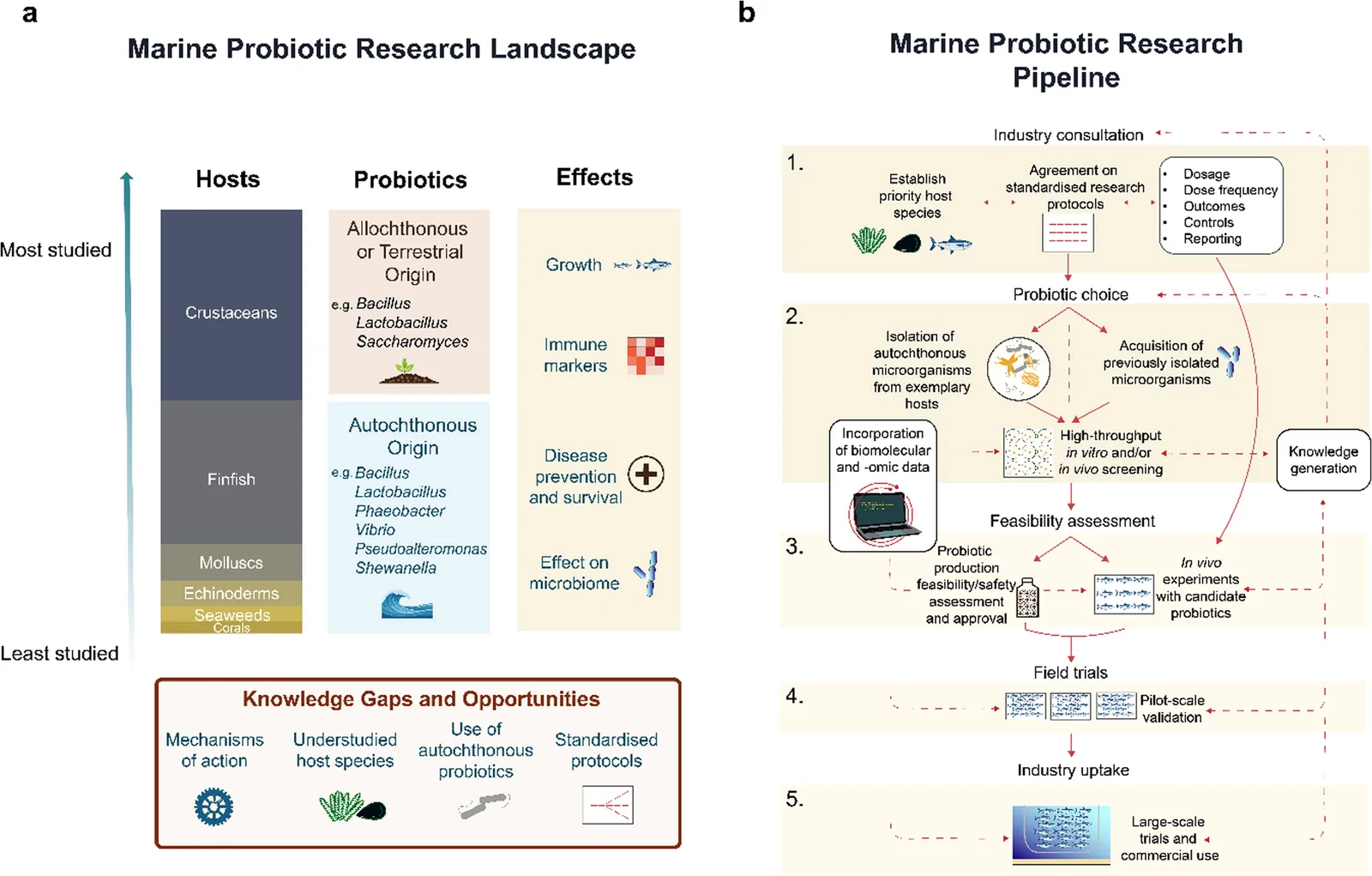
An overview of the status and future direction for marine probiotic research. **a**: Marine probiotic research landscape showing most to least studied host groups, sources of probiotics used in marine research and example bacteria genera, and key effects studied. Knowledge gaps and opportunities for research are also highlighted in the box at the bottom of the figure and can include (i) understanding probiotic mechanisms of action e.g., immune stimulation or competitive exclusion to increase host survival or prevent disease, digestive enzyme or precursor production for growth enhancement or feed conversion efficiency, and epigenetic changes that may influence both host growth and immunity, (ii) opportunities for probiotic research to assist in the production of understudied host species including seaweeds and molluscs, (iii) opportunities to better understand and utilise autochthonous probiotics across all host types, and, (iv) standardising protocols within specific research and aquaculture sectors to allow for better cross-study comparisons and meta-analyses. **b**: Recommended marine probiotic research pipeline that addresses key challenges and opportunities. Panel 1: Researchers work with industry to establish priority aquaculture species, develop standardised host or industry-specific protocols including dosage, delivery and outcomes to be measured. This framework informs the decision to discover new probiotic species from host organisms or use previously isolated probiotics and informs high-throughput testing protocols (panel 2). Following successful small-scale trials, feasibility studies of both probiotic production and scaled-up in vitro testing ensure probiotic safety and utility, in conjunction with gaining approval for use or ensuring adherence to local biosecurity laws in regions in which they may be applied (panel 3). Successful probiotics at this scale can be validated in field trials and larger pilot studies (panel 4) and are then used in commercial aquaculture operations (panel 5). At each stage, biomolecular and -omic data including genomics, proteomics, metabolomics and transcriptomics support laboratory and field research and help inform future decisions. Results from well-designed research allow for knowledge generation which can then be used for meta-analyses, evaluation and to inform future research and applications

Despite global growth in mariculture, host microbiome manipulation and the development of marine probiotics has traditionally lagged that of terrestrial systems. Our analysis of the publicly available research shows current understanding of marine probiotics is limited in scope, with underrepresentation of some hosts and probiotic species, methodological inconsistencies and a lack of standardised protocols hindering the ability to effectively compare across studies (Fig. 3a). Here we discuss these gaps and identify the research priorities that will be required to ensure marine probiotics reach their potential.

### Geographic and Taxonomic Biases

Assessment of the published literature suggests that the location of probiotic research does not align with geographic distribution or economic value of aquaculture production. Although Asia produces most aquaculture products, much of the research originates in Europe (supplementary table S1). This imbalance may reflect disparities in research funding, access to research infrastructure, publication of non-indexed or confidential industry data, or simply a focus on research outside of probiotics (Naylor et al. 2021, Palcullo et al. 2021, Gerbin and Drnovsek 2020). Directing research efforts towards regions with high dependence on aquaculture, particularly lower-income countries, would support capacity building and increase equity and resilience in the global blue economy (Naylor et al. 2021, Belton and Thilsted 2013, Bennett et al. 2019).

At the taxonomic level, research into different host species appears unrelated to their production quantity or value (supplementary table S1). Crustaceans and finfish dominate probiotic research, while molluscs and seaweeds remain underrepresented even when accounting for value and despite their major contribution to global aquaculture. These discrepancies may reflect differences in research priorities, funding accessibility, historical research momentum or disease pressures across aquaculture sectors.

Considering the global growth of the mollusc and seaweed aquaculture sectors, solutions to increase productivity and disease management is critical. In molluscs, hatchery output is repeatedly curtailed by vibriosis and other multifactorial syndromes such as Pacific Oyster Mortality Syndrome, yet antibiotic use is discouraged because of observed antibiotic resistance in pathogens (Dubert et al. 2017). This leaves probiotic and microbiome-based interventions as a possible alternative to antibiotic use. In seaweeds, diseases such as ice-ice syndrome and epiphyte outbreaks affect biomass yield and product quality (Sugumaran et al. 2022), and open-water cultivation of most seaweeds renders chemical and antibiotic treatments largely unworkable and environmentally risky. Probiotic inoculation and other microbiome-based approaches, including holobiont-focused breeding and priming of early life stages (Vadstein et al. 2018, Rodino-Janeiro et al. 2026, Thatcher et al. 2025) are therefore emerging as tools that address both concerns around chemical and antibiotic use and mariculture production constraints (Li et al. 2022).

### Selection of Appropriate Probiotic Strains

*Bacillus* and *Lactobacillus* dominate probiotic research in marine organisms, reflecting their historical use and status as Generally Recognised as Safe (GRAS) organisms for terrestrial and human applications without necessarily considering their suitability in marine hosts (Cutting 2011, Azad et al. 2018, Tsotetsi et al. 2022, Ringø et al. 2020, Hasan and Banerjee 2020, Martínez Cruz et al. 2012). Autochthonous probiotics, many of which are from genera other than *Bacillus* and *Lactobacillus*, are generally considered superior to allochthonous microorganisms, as they are already adapted to their intended host and local environment, and have demonstrated in vivo benefits (Doan et al. 2020, Lee et al. 2024, Lazado et al. 2015, Goulden et al. 2012). Their use also negates the risks (real or perceived) that foreign microorganisms may pose to new environments (Vargas-Albores et al. 2017, Moore et al. 2022).

Many lactic acid bacteria have been isolated from marine organisms and have potential as probiotics (Čanak et al. 2024), however genera including *Pseudoalteromonas*,* Halomonas*, *Shewanella*, and *Phaeobacter* may be more suitable in a marine context. These bacterial genera are naturally associated with marine hosts and are known to produce bioactive compounds and form biofilms (Holmström and Kjelleberg 1999, Sonnenschein et al. 2021, Kaye and Baross 2000, Ye and Chen 2021). These same genera were routinely isolated from a variety of marine hosts (see supplementary Excel file, Table 1), suggesting that they can be incorporated into host microbiomes (Verschuere et al. 2000). For example, *Shewanella putrefaciens* Pdp11, originally isolated from the gilthead seabream *S. aurata* (Chabrillón et al. 2005), showed antimicrobial activity against known pathogens and adhesion to host skin or intestinal mucus. This strain was subsequently used as an effective probiotic in the same host in several publications (Chen et al. 2020, Cordero et al. 2015, Esteban et al. 2014, Cordero et al. 2016). Marine-specific bacteria such as *Shewanella* sp. may prove more effective in marine contexts, however in some regions their use may require navigating the complex global policy landscape on probiotic use (Leistikow et al. 2022). With coordinated research and regulatory support, marine-specific probiotics could offer a promising long-term strategy for sustainable aquaculture.

While probiotic consortia are routinely reported to outperform single isolates, the mechanisms behind these synergistic effects remain unclear. Some publications investigating consortia tested each individual probiotic species within the consortia (Licona-Jain et al. 2022, Wang et al. 2019). For example, Wang et al. (Wang et al. 2019) used four probiotic microorganisms as single isolates and in different combinations as consortia in *L. vannamei*. Compared to single isolates or the control diet, consortia using either all four isolates, or a combination of *Lactobacillus pentosus *BD6 and *B. subtilis *E20, significantly improved shrimp growth and survival after challenge with pathogen *V. alginolyticus*. The consortia-treated shrimp also had enhanced immune responses. Other publications showed similar positive effects using consortia (Banerjee et al. 2010, Bernal et al. 2017, Pinoargote et al. 2018, Li et al. 2024, Nimrat et al. 2011, Shadrack et al. 2022). However, many publications only report on the benefit of a consortium compared to uninoculated controls, rather than individual members of the consortia. For example, Goda et al. (Goda et al. 2018) demonstrated enhanced growth, immune markers, and reduced *Vibrio* load in European seabass (*D. labrax*) treated with a single commercial probiotic and a commercially available multi-strain consortium, and their combination, but the members of the commercial consortium were not tested separately. This does not negate the efficacy of the consortium, but it does make it difficult to conclude if the probiotic benefit is due to a synergistic effect of the consortium or the impact of one or more individual strains. Understanding the interactions between probiotic members and between probiotics and host microbiota is essential for designing effective mixed-species probiotics. Consortia may also prove effective in multiple host species, but care should be taken to test each host specifically because evidence suggests an effective consortium in one host may not have the same benefits in another (Garcia et al. 2019). Future research should routinely experimentally separate the effects of individual and combined strains and assess how environmental conditions influence their collective performance.

Overall publications reported similar frequencies of positive outcomes for the host regardless of whether probiotics were autochthonous or allochthonous, and irrespective of the host life stage. However, descriptive differences emerged in studies using terrestrial versus marine probiotics. Crustacean and finfish publications using terrestrially derived probiotics reported less positive-only outcomes compared to those publications using marine-derived probiotics (73.2% vs. 84.8% in crustaceans, and 68.5% vs. 80.7% in finfish). A similar pattern was evident for molluscs and echinoderms, however, further investigation will be required to determine whether these patterns represent genuine differences in the reported efficiency of probiotic performance.

### Inconsistent Methodologies and Poor Mechanistic Understanding of Mariculture Probiotics

Despite overall positive findings, comparing probiotic effects across publications is challenging due to substantial methodological heterogeneity. Probiotic concentration, delivery method, and study design vary widely, with limited control for dietary or nutritional confounders (Liñan-Vidriales et al. 2021, Aguilar-Macías et al. 2010, Salehi et al. 2023, Siddik et al. 2022, Zaineldin et al. 2021, Gamboa-Delgado et al. 2016, Niu et al. 2021, Xu et al. 2019, Dawood et al. 2021, Zhao et al. 2018, Ashokkumar et al. 2016, Golder et al. 2022). The range of application methods – via feed, immersion or water treatment – likely contributes to variable outcomes. Similar inconsistencies were identified in a recent meta-analysis of shrimp probiotic research, with 90% of studies excluded due to inconsistent experimental design (Golder et al. 2022). A standardised approach to probiotic research and reporting will increase confidence in probiotic use and aid in understanding probiotic mechanisms of action.

Even as the use of molecular tools increases, mechanisms by which probiotics exert their effects remain poorly defined. Fewer than one-third of studies linked improved growth or survival with specific mechanistic measurements such as digestive enzyme activity, immune modulation, or microbiome change, highlighting that most research reports on outcomes rather than underlying mechanisms. In studies with microbiome analyses, researchers inferred causation such as competitive exclusion or pathogen suppression based on correlative microbiome data rather than direct evidence, limiting mechanistic interpretation. Moreover, the use of widely accessible and cost-effective 16S ribosomal RNA (rRNA) gene sequencing technology can have limitations and biases (Temperton et al. 2009, Hardoim et al. 2014) that are not always acknowledged. Both short-read (< 500 bp) and full length 16S rRNA gene sequencing cannot always achieve species-level differentiation between marine probiotic microorganisms, autochthonous symbionts, and potential pathogens (King et al. 2019, Pascual et al. 2010). As a result, the exclusion of microorganisms based solely on genus-level identification may also overlook beneficial taxa (Madison et al. 2022, Sawabe et al. 2013).

Amplicon primers that target specific microbial taxa offer one avenue for improved resolution. For example, Vibrio-targeted amplicon primers, already proven to show greater species-level resolution for pathogen surveillance (King et al. 2019), could similarly distinguish pathogenic, commensal, and probiotic vibrio strains in aquaculture microbiome studies. Similar approaches could be used to achieve greater resolution of other microbial genera of interest, and the growing adoption of long-read sequencing as costs decrease will allow for better resolution of both natural host microbiomes and changes induced by probiotics. Targeted sequencing methods and controlled mechanistic experiments could help clarify whether positive outcomes stem from immune modulation, competitive exclusion, metabolite production, or other host–microbe interactions.

The need for more rigorous and standardised approaches in probiotic research has been recognised for decades. Verschuere et al. (Verschuere et al. 2000) proposed guidelines for probiotic research in aquaculture 25 years ago, however we found many concerns raised then remain in the current research landscape. Extensive research guidelines on selection of probiotics, reporting and study design exist in human probiotic research (e.g. (FAO/WHO 2002, Shane et al. 2010, Hill et al. 2014)). Similar guidelines tailored to marine systems could steer future biotechnology research that implements adequate controls, randomisation, dosage and other important parameters to enable cross-study comparison not just between different probiotics but also between hosts (Fig. 3b). We propose a 5-stage marine probiotic research pipeline, incorporating (1) sector-specific standardised protocols to ensure studies can be adequately replicated and compared (2) high-throughput testing of previously isolated and host-associated microorganisms from exemplary hosts, combined with multi -omic approaches, (3) feasibility assessment testing safety, production and small-scale in vivo trials, (4) field trials with pilot scale validation, (5) large scale trials and industry uptake. At each stage, knowledge generation and industry consultation feed back into earlier steps for future research.

## Conclusion

This review shows that while probiotics hold considerable promise for mariculture, the field remains constrained by its reliance on concepts and microorganisms from terrestrial systems. We argue that this legacy contributes to three main shortcomings in marine probiotic research: (1) a continued focus on finfish and crustaceans at the expense of other ecologically and economically important hosts such as molluscs and seaweeds, (2) a reliance on *Bacillus* and *Lactobacillus* that reflects their GRAS status and historical precedent rather than any demonstrated suitability for marine organisms, and (3) a lack of standardised, mechanism-focused studies.

Addressing these concerns will be essential if marine probiotics are to reach their potential. Prioritising autochthonous, marine-derived probiotic strains will minimise biosecurity risks and ensure that probiotics are fit for purpose in their intended hosts. Standardised, sector-specific research frameworks, such as the pipeline we propose, would allow results to be compared across studies and hosts, and support meta-analyses. Combining standardised trials with mechanistic and multi-omic approaches would move the field beyond correlative microbiome studies towards a mechanistic understanding of probiotic benefits, facilitating the rational design of probiotic consortia.

Future advances will depend not only on improved probiotic strain selection, but also on methodological, geographical and application-focused developments. Taxon-targeted amplicons, together with the growing adoption of long-read sequencing as costs decline, will improve the ability to distinguish probiotic strains from closely related commensals and pathogens at a much finer taxonomic resolution than 16S rRNA gene sequencing alone. Directing more mariculture probiotic research towards the lower-income, aquaculture-dependent regions that produce most of the world’s aquatic biomass would also help promote greater equity and resilience across the global blue economy. Beyond food production, microbiome-based interventions are showing early promise in improving the environmental resilience of coastal ecosystem engineers including corals, seagrasses and kelps, and holobiont-focused strategies including priming of early life stages (Vadstein et al. 2018, Rodino-Janeiro et al. 2026, Thatcher et al. 2025) may extend the benefits of probiotics towards broader marine restoration efforts (Garcias-Bonet et al. 2024, Corinaldesi et al. 2023).

Marine probiotics have the potential to transform both sustainable aquaculture and broader marine ecosystem management. Realising this opportunity requires mechanistically informed, marine-centred research that enables probiotics to be developed and applied effectively, safely and with confidence.

## Supplementary Information

Below is the link to the electronic supplementary material.

Supplementary Material 1 (XLSX 351 KB)

Supplementary Material 2 (DOCX 121 KB)

## Data Availability

All data collected from each publication used in the scoping review is available in the Supplementary Materials.

## Declarations

This research was supported by the Commonwealth through an Australian Government Research Training Program Scholarship [DOI: https://doi.org/10.82133/C42F-K220]. The authors acknowledge the use of OpenAI GPT-4.5 2025 to edit data visualisation scripts and GPT-5.3 2026 as a support tool for language refinement and copy editing of specific sections of the text.
