# Supplementary material for: Mariculture Probiotics: A Scoping Review of Research Trends, Knowledge Gaps and Opportunities
Source: Mar Biotechnol (NY). 2026 Sep 4;28(5):152. doi: 10.1007/s10126-026-10701-7 (PMC13545048; doi:10.1007/s10126-026-10701-7)
Supplement: Supplementary file 1 [file 10126_2026_10701_MOESM2_ESM.docx]

**Supplementary Figure 1**: Global distribution of mariculture probiotic publications. Countries are shaded by the number of primary studies (n = 892) and attributed to the country where the study was conducted, for records to 25 May 2025. Grey indicates countries with no recorded publications.

**Supplementary Table 1**: Publication relative to production volume and economic value across aquaculture sectors*.

| **Host Type** | **Production (×1000 tonnes)** | **Value (billion USD)** | **Publications** | **Publications per billion USD** | **Publications per million tonnes** |
| --- | --- | --- | --- | --- | --- |
| Echinoderm | 344 | 2.67 | 53 | 19.9 | 154.1 |
| Crustacean | 8,259 | 48.84 | 401 | 8.2 | 48.6 |
| Finfish | 8,130 | 45.18 | 303 | 6.7 | 37.3 |
| Mollusc | 20,283 | 40.51 | 77 | 1.9 | 3.8 |
| Seaweed | 38,777 | 19.71 | 33 | 1.7 | 0.9 |

* Data from 2024 Food and Agriculture Organisation of the United Nations [FAO] aquaculture species quantity and value [1,2]. Note that corals are not included in the data and so were excluded from this analysis. Publications conducted on multiple host types were counted for each host type.

**References**

1. Food and Agriculture Organization of the United Nations, 2024. "Global aquaculture production quantity (1950 - 2022)." Accessed online: https://www.fao.org/fishery/statistics-query/en/aquaculture/aquaculture_quantity

2. Food and Agriculture Organization of the United Nations, 2024. "Global aquaculture production value (1984 - 2022)." Accessed online: https://www.fao.org/fishery/statistics-query/en/aquaculture/aquaculture_value
